# Hybrid Network with Attention Mechanism for Detection and Location of Myocardial Infarction Based on 12-Lead Electrocardiogram Signals

**DOI:** 10.3390/s20041020

**Published:** 2020-02-14

**Authors:** Lidan Fu, Binchun Lu, Bo Nie, Zhiyun Peng, Hongying Liu, Xitian Pi

**Affiliations:** 1 Chongqing University-University of Cincinnati Joint Co-op Institute, Chongqing University, Chongqing 400030, China; fuln@mail.uc.edu (L.F.); lubu@mail.uc.edu (B.L.); 2 Key Laboratory of Biotechnology Science and Technology, Ministry of Education, College of Bioengineering, Chongqing University, Chongqing 400030, China; 20165686@cqu.edu.cn; 3 State Key Laboratory of Power Transmission Equipment & System Security and New Technology, Chongqing University, Chongqing 400030, China; zhiyun.peng@cqu.edu.cn

**Keywords:** myocardial infarction, electrocardiogram, attention mechanism, convolutional neural network, bidirectional gated recurrent unit

## Abstract

The electrocardiogram (ECG) is a non-invasive, inexpensive, and effective tool for myocardial infarction (MI) diagnosis. Conventional detection algorithms require solid domain expertise and rely heavily on handcrafted features. Although previous works have studied deep learning methods for extracting features, these methods still neglect the relationships between different leads and the temporal characteristics of ECG signals. To handle the issues, a novel multi-lead attention (MLA) mechanism integrated with convolutional neural network (CNN) and bidirectional gated recurrent unit (BiGRU) framework (MLA-CNN-BiGRU) is therefore proposed to detect and locate MI via 12-lead ECG records. Specifically, the MLA mechanism automatically measures and assigns the weights to different leads according to their contribution. The two-dimensional CNN module exploits the interrelated characteristics between leads and extracts discriminative spatial features. Moreover, the BiGRU module extracts essential temporal features inside each lead. The spatial and temporal features from these two modules are fused together as global features for classification. In experiments, MI location and detection were performed under both intra-patient scheme and inter-patient scheme to test the robustness of the proposed framework. Experimental results indicate that our intelligent framework achieved satisfactory performance and demonstrated vital clinical significance.

## 1. Introduction

Myocardial infarction (MI), as one of the most prevalent cardiovascular diseases worldwide, commonly emerges when the coronary artery is occluded by thrombus. It is estimated that the annual incidence of MI is 605,000 new attacks and 200,000 recurrent attacks in the United States [1]. In fact, MI is also described as silent heart attack and most patients suffer from MI without awareness. Even worse, acute MI occurs rapidly and unexpectedly with a high mortality rate. Therefore, early diagnosis and timely treatment are of utmost significance to guarantee the life safety of MI patients.

Electrocardiographic (ECG) can be employed to recognize MI [2], which serves as the most popular diagnostic tool for its convenience, non-invasiveness and low cost. ECG records the electrical signals generated by the heart muscle fibers during the alternate contraction and relaxation of the heart chambers [3]. A normal ECG is characterized by the cardiac cycle sequence, and each cycle mainly contains P, QRS, and T waves. In general, ECG consists of 12 leads (I, II, III, aVR, aVL, aVF, and V1–V6) that reflect the heart in various regions and perspectives. The location of MI can be detected by the alterations among different leads [2]; therefore, it is essential to take more leads into account in the diagnosis of MI. However, it is strenuous and time-consuming for the trained physicians to evaluate every lead precisely. Moreover, because of ECG individualized polymorphism, the diagnostic criteria are perplexing and complicated to follow [4]. The ST-segmental elevation is one of the diagnostic indicators of MI [2], but even experienced cardiologists may only identify 82% of this indicator among MI subjects [5]. A computer-aided diagnosis (CAD) system can exceed the limitations of manual inspection of ECG signals by its rapid, objective, and reliable analysis [6]. Hence, effective diagnosis of MI with 12-lead ECG signals analyzed by CAD system is advantageous and preferable.

Various frameworks have been proposed and developed in the CAD system for MI detection and location. Most of the studies follow the procedure of feature extraction, feature selection, and classification. Conventionally, the process of feature extraction is manual operation and requires solid domain expertise. Several characteristic values can be extracted from ECG morphology as relevant features of MI, such as ST deviation and T wave amplitude [7]. However, most of morphological features are heavily dependent on the accuracy of ECG wave delineation. To mine additional information, wavelet transform, principal component analysis (PCA), empirical mode decomposition, random projections, hidden Markov model, and reproducing kernel Hilbert space are employed to extract the representative features [8,9]. After feature extraction or selection, diverse classifiers are developed to discriminate between MI and healthy controls (HCs) through the obtained features. Additionally, multi-classification classifiers are applied to localize different types of MI. The classifiers can be typically categorized into traditional thresholding methods [10] and machine learning algorithms. Conventional machine learning classifiers include K-nearest neighbor [11], random forest [12], and support vector machine [13]. Although the above off-the-shelf methods work well, they still have obvious defects and limitations. In essence, the feature extraction and classification are two separate modules with substantially different parameters and complexity. It is hard to determine whether the information is fully excavated or redundantly used, which exerts adverse impact on the subsequent classification. Furthermore, specific feature extraction algorithms have unconvinced robustness under different influence factors, such as age, gender, and acquisition equipment. Therefore, an automatic and end-to-end framework that integrates effective feature extraction and classification processes is required to improve the effectiveness of MI diagnosis.

In recent decades, deep learning methods, including convolutional neural network (CNN), gated recurrent unit (GRU), attention mechanism, and autoencoder, have been widely and superbly applied to analyze biomedical signals [14,15,16]. Instead of separate feature extraction and classification processes, deep learning architectures automatically extract critical features required for classification from vast samples [17]. Furthermore, CNN and GRU are two typical end-to-end learning paradigms with multiple levels of representation and especially suitable for discovering the spatial and temporal characteristics in high-dimensional data [18]. To alleviate the disadvantages of conventional frameworks, deep learning methods are exploited in MI diagnosis continuously and rapidly[19,20,21,22,23,24]. To a large extent, new research lays the foundations for the development of deep learning frameworks that make full use of 12-lead ECG signals.

Although there exists plenty of research on MI diagnosis, several detailed issues are still without due consideration. Basically, most studies only utilized the single lead of ECG, but the rest should also be taken into account. It is more in conformity with the authentic rules of MI diagnosis to consider 12-lead ECG records [24]. Secondly, thus far, importance evaluation and weighted combination of each lead in MI diagnosis have been sparsely investigated. Even though the authors of  [22,23,24,25] considered 12 leads simultaneously, each lead contained distinctive and complementary information that deserved different and separate processing rather than identical treatment. Thirdly, only a few researchers considered the inter-patient scheme on the Physikalisch-Technische Bundesanstalt (PTB) dataset. Since the individual variation exists in different patients, inter-patient scheme is closely relevant to clinical practice and applications. On the contrary, intra-patient scheme cannot substantiate the feasibility and adaptability of the model and may even bring about overly sanguine diagnosis.

To address the aforementioned limitations, a novel, practical, and medical-grade framework is proposed for the detection and location of MI. More precisely, the main contributions of this study are listed as follows.

1. A novel multi-lead attention (MLA) mechanism integrated with CNN and bidirectional gated recurrent unit (BiGRU) framework (MLA-CNN-BiGRU) is proposed. The parallel deployed CNN and BiGRU modules are innovatively utilized to extract features to detect and locate MI via 12-lead heartbeat signals. As far as we know, this fills the gap of applying deep learning methods to automatically extract spatial and temporal features from 12-lead ECG signals in MI diagnosis. The proposed feature extraction method paves a new way for feature engineering.
2. The MLA is developed by the designed activation function. The proposed attention mechanism measures and exploits the contribution of each lead to boost the diagnostic performance. Existing studies mainly focus on manual selection of leads or treat all the leads equally with repeated and redundant information. With the proposed model-based approach, this study serves as a preliminary exploration on the importance evaluation of each lead for MI detection and location.
3. Different leads are interrelated and correlated. It is essential to fully exploit available features to enhance the performance. To our knowledge, it is the first time to adopt 2D-CNN to extract spatial features based on multi-lead fusion in MI diagnosis. Three different convolutional kernels are innovatively applied to extract correlation and regional features among different leads.
4. MI detection and location under intra-patient and inter-patient schemes are all performed to test the robustness of MLA-CNN-BiGRU. In addition, elaborate and exhaustive ablation experiments are carried out to verify the effectiveness of the framework. Experimental results indicate that the proposed intelligent framework achieves satisfactory performance and demonstrates vital clinical significance.

## 2. Related Work

Before introducing the proposed hybrid deep learning framework, background information of attention mechanism, CNN, and GRU is illustrated as guidance.

### 2.1. Attention Mechanism

Inspired by the efficient allocation of limited resources by the human brain, attention mechanism is widely applied to emphasize the most valuable information in visual image recognition [26] and natural language processing [27]. Since redundant information is time- and resource-consuming in the data processing, self-attention mechanism [28] is proposed for sequential models to calculate the weights for different features. Generally, self-attention is deployed on the outputs of GRU- or CNN-based sequential models [29].

Recently, attention mechanism has been popular in clinical diagnosis. Deep fusional attention network was adopted to extract elaborate features from biological signals in seizure detection and sleep stage classification [16]. In MI diagnosis, the heartbeat-attention mechanism was introduced to automatically weight the difference between unlabeled heartbeats [22]. Furthermore, the attention mechanism has strong interpretability. Its ability to evaluate importance and contribution can be implemented not only for feature extraction, but also for multi-channel screening.

### 2.2. Convolutional Neural Network

CNN is the most established architecture in image recognition field, which is enlightened by the natural visual perception mechanism of creatures [30,31]. Typically, CNN consists of three types of stacked layers combined with a series of manipulations. Convolutional layers apply convolutional kernels to learn different spatial feature maps of the input data. Pooling layers reduce the dimensionality of the feature maps from convolutional layers with shift-invariance [32]. Fully connected layers perform the final classification or prediction. Batch normalization manipulation can improve the training rates by preventing the phenomenon of internal covariate shifting [33]. Dropout manipulation can reduce overfitting by avoiding complex co-adaptations on the training data [34]. Activation functions introduce nonlinearities to neural networks. Typical activation functions are sigmoid, tanh, and rectified linear unit (ReLU) [32]. The loss function defines the difference between the real value and the predicted value. During the training process, the optimizer minimizes the loss function, and the best fitting parameters can be obtained.

In the medical field, there has been a rapid surge of applications of CNN among radiology  [31] and physiological signals [14]. Researchers have applied CNN by treating ECG signals as the 1D image in the diagnosis of MI [19,35,36,37]. Deep CNN was applied to automatically diagnose MI through one single lead and attained good performance [19,35]. Baloglu et al. [36] achieved impressive results based on CNN model with all the 12 leads. Multiple-feature-branch 1D CNN was created to take full advantage of 12 leads [37]. Multi-lead residual neural network was proposed, and three residual blocks were designed to capture remarkable features by convolutional layer through 1D convolutional kernel [24]. Additionally, sub 2D CNN structure extracted different feature representation with shared 1D convolutional kernels among four leads during MI detection [20]. In essence, 1D CNN only focuses on the features within the single lead. Although the sub 2D CNN was applied, the feature map was still generated based on the shared 1D convolutional kernel inside the same lead. Therefore, the powerful feature extraction ability of 2D CNN through multi-lead convolutional kernels remains further development in the diagnosis of MI.

### 2.3. Gated Recurrent Unit

Recurrent Neural Network (RNN) is widely used in the processing of time series data due to its ability to memorize sequential information. RNN implements a recursive task with the output being dependent on all the historical information [17]. However, the total memory capacity is restricted in standard RNNs. Long Short-Term Memory (LSTM) [38] is designed to avoid sacrificing too much information in learning long-term dependencies by addressing the vanishing gradient problem. In LSTM, a memory block continuously transmits and renews memory by three gates: the input, output, and forget gates. The input gate identifies what new information is important and needs to be reserved in the previous state. The output gate determines what information is conveyed to the next state. The forget gate identifies what relevant information needs to be retained in the previous state. GRU [39] is created as an enhanced variant of LSTM that can extract features selectively through a reset gate and an update gate. Compared with LSTM, GRU has no cell state and straightforwardly uses hidden state for the transmission of information. The reset gate of GRU is utilized to determine how much previous information requires to be forgotten. The update gate determines what previous information to keep and what new information to merge. Apart from optimizing the internal structure, GRU can be further improved by taking all the previous and subsequent context information into consideration. Therefore, bidirectional GRU that is integrated by two GRU layers [40] is proposed. BiGRU processes information in backward and forward directions and is therefore able to exploit both the past and the future information.

In processing biomedical signals, BiGRU has been successfully applied for human emotion classification through continuous electroencephalogram signals [41], and human identification through ECG based biometrics [42]. ECG signal is a typical kind of time series data, and LSTM has been effectively applied in MI diagnosis [21,22,23]. GRU architecture can achieve performance comparable to or even superior than LSTM [42], but its potential has been rarely investigated in MI diagnosis thus far.

## 3. Dataset and Pre-Processing

The ECG data utilized in this study were from PTB dataset provided by the German National Metrology Institute [43]. The PTB dataset contained 549 records from 290 subjects. Each record was obtained by synchronous acquisition of 15 leads, including conventional 12 leads ECG and the 3 Frank signals. The sampling frequency of electrical signals in PTB dataset was 1000 Hz. In the dataset, 148 MI patients (368 records) and 52 healthy volunteers (80 records) were collected. The ECG signals of 148 MI patients were identified as ten different types of MI, but only five categories were selected in MI location. Specifically, 314 records were used for MI location, including 47 records of anterior MI (AMI), 43 records of antero-lateral MI (ALMI), 79 records of antero-septal MI (ASMI), 89 records of inferior MI (IMI), and 56 records of inferolateral MI (ILMI).

The pre-processing of ECG signals included denoising, removing baseline drift and data segmentation. To eliminate the magnitude difference between different records, data standardization transformed all input data into values within [−1,1]. Daubechies 6 (DB6) wavelet basis function [44] was applied to eliminate noise and remove baseline drift. Additionally, Pan–Tompkin algorithm [45] was employed to segment or select the pre-processed ECG signals by QRS-wave detection. In detail, 250 sample points were selected before the QRS-peak point and 400 sample points were chosen after the QRS-peak point, which formed a heartbeat segment composed of 651 points. Moreover, the first and last heartbeats were removed from each ECG signal record. Table 1 demonstrates the data distribution of 12-lead heart beats in this study.

## 4. Methodology

**Figure 1:**
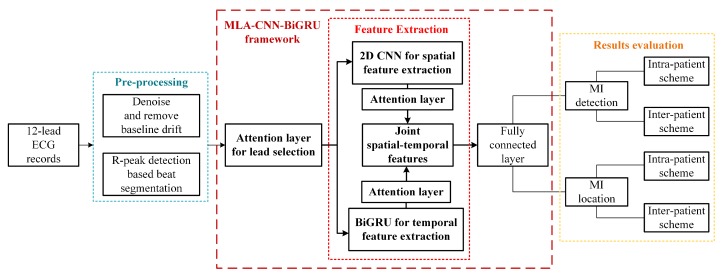
Overall scheme of the research.

The framework of hybrid neural network is comprised of three sub-modules, as shown in Figure 1. Firstly, pre-processed data are inputted into the MLA-CNN-BiGRU framework. An attention layer is trained to determine the importance of each lead. After adaptive selection, CNN is applied to extract spatial features. Thereinto, features are weighted and integrated via attention mechanism. Simultaneously, BiGRU with feature integration attention mechanism mines optimal features in the temporal dimension. Ultimately, the spatial and temporal features from two modules are joined and fed into the fully connected layer for classification.

### 4.1. Multi-lead Attention Module

**Figure 2:**
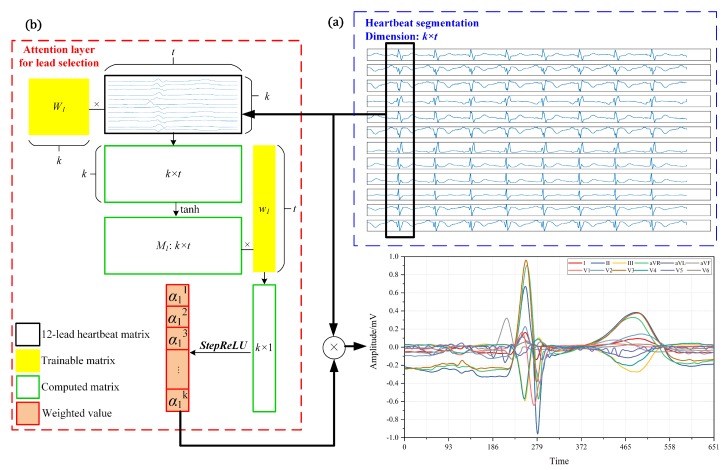
Schematic diagram of multi-lead attention (MLA) mechanism: (**a**) heartbeat segmentation; and (**b**) MLA mechanism outputs the weighted heartbeat signals.

In a segmented heartbeat with 12 leads, each lead reflects the heart condition from different perspectives. Undesired and unnecessary information could have a reverse impact on the training process, even limiting the maximum performance of the model. For this reason, the identification of effective input data is particularly important. However, treating all the leads equally could result in redundant information. Training neural networks with repetitive information is time-consuming and resource-wasting. When analyzing 12 leads for MI identification, not all leads make equal contributions. Therefore, the attention mechanism is elaborately employed to evaluate the significance of each lead. The attention mechanism shown in Figure 2 makes the weighted information of 12 leads more condensed and refined, thus facilitating the subsequent processing.

In this study, self-attention mechanism is modified to measure the importance of each lead. The proposed MLA, an extension of the conventional attention mechanism, can be used for lead selection through the designed activation function. The proposed MLA mechanism aims to heavily weight key leads and eliminate redundant leads. To achieve this purpose, a modified version of the activation function ReLU is therefore adopted.

(1) $$StepReLU\left(x\right)=\left\{\begin{array}{cc} \begin{array}{l} \;0\\ \;x\\ \;1 \end{array} & \begin{array}{l} \;(x<0)\\ \;(0\leq x\leq 1)\\ \;(x>1) \end{array} \end{array}\right.$$

As shown in Equation (1), the StepReLU is created to simulate the step function. After the weight is activated by the StepReLU, its value is distributed between zero and one. In this way, the crucial leads could be entirely retained and leads of no use could be completely abandoned. The remaining leads are assigned with partial weights. The ordinary step function is either zero or one, and its derivative is zero, therefore it cannot be applied to train neural networks. StepReLU can be used in the back-propagation algorithm and serves a similar purpose as a step function. Moreover, the proposed activation function solves the issue that maximum values after traditional ReLU activation are uncontrolled.

The implementation process of MLA can be summarized by Equations (2) and (3).

(2) $$M_{1}=\tanh\left(W_{1}L+b_{1}\right)$$

(3) $$\alpha_{1}=StepReLU\left(M_{1}w_{1}\right)$$

(4) $$X=\alpha_{1}⊗L$$

where $L^{T}=\left[l_{1},l_{2},\ldots ,l_{k}\right]\left(l\in R^{t},L\in R^{k\times t}\right)$ is the input heartbeat sample with 12 leads and 651 time points (k=12,t=651). $W_{1}\in R^{k\times k}$ is a trainable parameter matrix. $w_{1}\in R^{t}$ is the parameter vector and $b_{1}\in R^{k}$ is the bias term. Function tanh(·) denotes hyperbolic tangent function. After the computation, the vector $\alpha_{1}\left(\alpha_{1}\in R^{k}\right)$ represents the importance of each lead. Finally, $X^{T}=\left[x_{1},x_{2},\ldots ,x_{k}\right]\left(x\in R^{t},X\in R^{k\times t}\right)$ shown in Equation (4) is the 12-lead signal after selection, where the self-defined multiplication ⊗ is $x_{i}={\alpha_{1}}^{i}\cdot l_{i}\left(i\in \left[1,2,\ldots ,k\right]\right)$. Through MLA mechanism, *L* is transformed into *X*, which serves as the input for subsequent feature extraction.

### 4.2. CNN with Attention Mechanism for Spatial Feature Extraction

**Figure 3:**
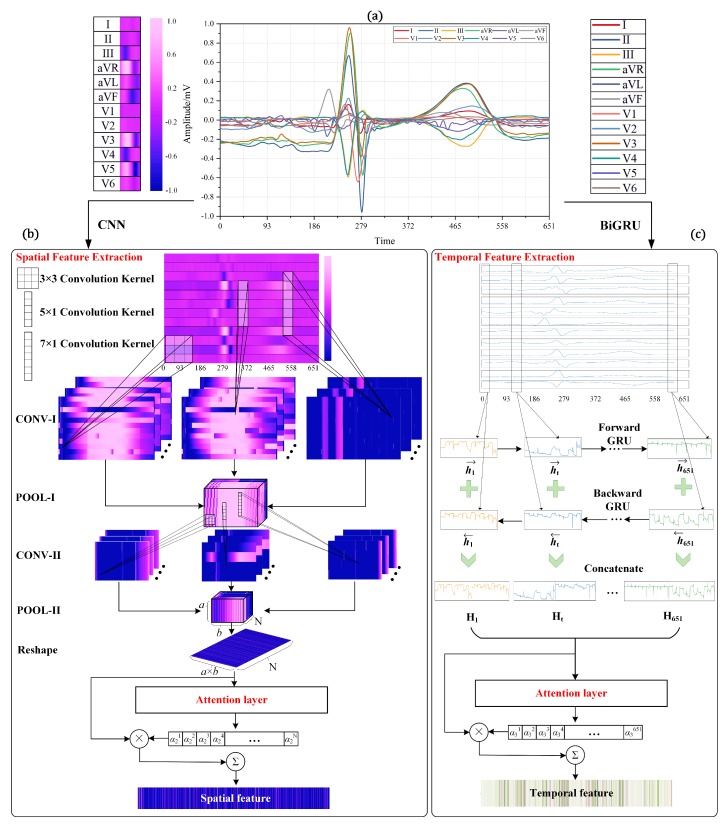
Feature extraction process after lead selection: (**a**) 12-lead heartbeat signal after lead selection; (**b**) spatial feature extraction process by CNN module; and (**c**) temporal feature extraction process by BiGRU module.

As a feature extraction module with the ability to identify the optimum spatial features for diagnosis, CNN is combined with attention mechanism to form one branch of the hybrid framework. This module consists of two alternated convolutional and pooling layers, as well as an attention layer in the end, as shown in Figure 3b.

Different leads are interrelated and correlated, but each lead is one-dimensional, thus making 2D CNN inapplicable. Inspired by multi-sensor data fusion [46], we utilized time dimension as the horizontal axis and arranged 12 leads in vertical axis to convert one-dimensional signal into two-dimensional data. Therefore, each 12-lead beat sample has the size of 12×651. To enable 2D CNN to effectively mine useful spatial features, three different convolutional kernels, namely 3×3 kernel, 5×1 kernel, and 7×1 kernel, are innovatively applied to extract the correlated and regional features among different leads. In this way, the 5×1 kernel can consider five leads at a time. Similarly, 7×1 kernel takes the information of seven leads into account at the same time point.

#### 4.2.1. Convolutional Layer

In the convolutional layer of CNN, high-order information can be extracted though convolution and activation operation. The input data are convolved with a set of kernels with different shapes to generate discriminative feature maps for diagnostic representation. Then, the nonlinearity is introduced by element-wise activation function. As illustrated in Equation (5), the feature value $x_{i,j,n}^{m}$ is computed by the *n*th kernel at location (i,j) in the *m*th layer.

(5) $$x_{i,j,n}^{m}=f\left(W_{n}^{m^{T}}x_{i,j}^{m-1}+b_{n}^{m}\right)$$

where $x_{i,j}^{m-1}$ is the input patch centered at location (i,j) in the (m−1)th layer. $W_{n}^{m}$ and $b_{n}^{m}$ are the weight and bias term of the *n*th kernel filter (n∈[1,2,…,N]) in the *m*th layer, respectively. Each kernel generates one feature map through sliding data window with shared weight and bias parameters. There are *N* kernels in each layer, which means *N* feature maps can be generated as input to the next pooling layer. Activation function is denoted as f(·) to produce nonlinearity.

#### 4.2.2. Pooling Layer

To reduce the dimensions and improve the robustness of the learned feature maps, pooling layer is generally concatenated between two convolutional layers. Features in the local patches of input maps are compressed to more robust representation to achieve subsampling. Therefore, pooling layers possess shift-invariance to minor transformations in the input images [47]. Moreover, computation burden during the training process can be reduced. Considering each beat may vary in morphology and numerical values, pooling layers can alleviate the influence of these variations to enhance the robustness. Max pooling is one of the typical pooling operations, which computes the maximum values in the pooling windows. Max pooling is effective for retaining texture information [47]. It is applied in this study because the texture characteristics, such as the peak and fluctuation of heartbeat, could be reserved during the subsampling.

#### 4.2.3. Attention Layer for CNN

After the operation of convolutional and pooling layers, a series of feature maps is ultimately formed. If all the feature maps are directly concatenated for classification, the parameters in the fully connected layer are doomed to be vast and easy to be overfitted. Furthermore, the contribution of each feature map is not equal. In fact, some feature maps are redundant and unnecessary in classification and thus should have small weights. On the contrary, pivotal and discriminative feature maps deserve greater weights.

Compared with conventional CNN models that treat all the feature maps in the same manner, an attention layer is added on top of CNN to integrate different feature maps and form optimal spatial feature representation for classification. The calculation process of the weight vector $\alpha_{2}$ is shown in Equation (6) and the final spatial feature vector $f_{s}$ is obtained by Equation (7). The input $x_{n}^{\prime }\in X^{\prime }$ denotes the *n*th feature vector in the whole features $X^{\prime }=\left[x_{1}^{\prime },x_{2}^{\prime },\ldots ,x_{N}^{\prime }\right]$ generated from the last pooling layer. The activation function softmax(·) ensures that all calculated weights in the vector $\alpha_{2}$ add up to 1. $W_{2}$, $b_{2}$, $w_{2}$ are trainable parameters.

(6) $$\alpha_{2}=Softmax\left(w_{2}\tanh\left(W_{2}{X^{\prime }}^{T}+b_{2}\right)\right)$$

(7) $$f_{s}=\sum_{n=1}^{N}{\alpha_{2}}^{n}\cdot x_{n}^{\prime }$$

Therefore, CNN combined with attention mechanism can better characterize the spatial features from signal data. Additionally, the proposed CNN module pays more attention to the correlation of adjacent leads and integrates discriminative features more reasonably.

### 4.3. BiGRU with Attention Mechanism for Temporal Feature Extraction

The ECG signal is essentially a periodic signal with certain regularity. Therefore, the heart state corresponding to the current sampling value is not only related to the previous time point, but also related to the information of the subsequent time point. To efficiently learn the temporal correlation of ECG signals in each lead, BiGRU with attention mechanism is accordingly employed to further strengthen the performance of the general framework. BiGRU module is deployed in parallel with CNN module, and they conduct training and parameters updating together. In detail, BiGRU module consists of two parallel GRUs and an attention layer in the end, as shown in Figure 3c.

#### 4.3.1. BiGRU Neural Network

GRU is designed to improve the three-gate structure of LSTM by removing cell state and conflating the forget gate and input gate to an update gate. Therefore, GRU has fewer parameters and performs more efficiently. The calculation principle of GRU is defined in Equation (8).

(8) $$\begin{array}{l} z_{t}=\sigma \left(W_{xz}x_{t}+W_{hz}h_{t-1}+b_{z}\right)\\ r_{t}=\sigma \left(W_{xr}x_{t}+W_{hr}h_{t-1}+b_{r}\right)\\ {\tilde{h}}_{t}=\tanh\left[W_{xh}x_{t}+W\left(r_{t}*h_{t-1}\right)\right]\\ h_{t}=\left(1-z_{t}\right)*h_{t-1}+z_{t}*{\tilde{h}}_{t} \end{array}$$

where $z_{t}$ represents the update gate and $h_{t-1}$ denotes the output of the previous neuron. ${\tilde{h}}_{t}$ is the signal information learned at the present state after the reset gate $r_{t}$. $h_{t}$ represents the hidden state of the neuron. $W_{xz}$, $W_{hz}$, $W_{xr}$, $W_{hr}$, $W_{xh}$, and *W* are the corresponding weight matrices. $b_{z}$ and $b_{r}$ are the bias terms. Function σ(·) and tanh(·) represent the sigmoid function and hyperbolic tangent function. The symbol * denotes the element-wise multiplication.

To make full use of the past and future information, BiGRU is developed by containing a forward GRU layer and a backward GRU layer. The input $x_{t}\in R^{k}$ holds the information of 12 leads at the same time point *t*. During the training process, GRU cell iterates 651 times for each beat sample to capture the temporal features. The hidden vectors $\vec{h_{t}}$ and $\overset{\leftarrow }{h_{t}}$ can be extracted as forward and backward temporal features, which are calculated by Equation (9). Subsequently, hidden states from two directions are concatenated to generate the overall temporal features *H* composed of $H_{t}$, as shown in Equation (10).

(9) $$\begin{array}{l} {\vec{h}}_{t}=\vec{GRU}\left(x_{t}\right),t\in \left[1,T\right]\\ {\overset{\leftarrow }{h}}_{t}=\overset{\leftarrow }{GRU}\left(x_{t}\right),t\in \left[T,1\right] \end{array}$$

(10) $$H_{t}=con\left[\vec{h_{t}},\overset{\leftarrow }{h_{t}}\right]$$

#### 4.3.2. Attention Layer for BiGRU

There are 651 total hidden states formed after BiGRU. Meanwhile, each hidden state provides diverse information and exhibits different contribution for the final classification. Similar to the attention layer in the CNN module, another attention layer is introduced after the BiGRU layer, as illustrated in Equations (11) and (12). $W_{3}$, $b_{3}$, and $w_{3}$ are trainable parameters. Correspondingly, each temporal feature extracted by BiGRU is assigned with an appropriate weight and features are integrated into the final temporal feature $f_{t}$.

(11) $$\alpha_{3}=Softmax\left(w_{3}\tanh\left(W_{3}H^{T}+b_{3}\right)\right)$$

(12) $$f_{t}=\sum_{t=1}^{T}{\alpha_{3}}^{t}\cdot H_{t}$$

### 4.4. Merge and Classification

In the proposed framework, the last step concatenates the features extracted by the two modules and co-trains them for classification. The training procedure is detailed in Algorithm 1. The proposed CNN module and BiGRU module are employed as spatial and temporal feature learners, respectively. The spatial feature $f_{s}$ and the temporal feature $f_{t}$ learned from the beat sample are concatenated into a joint feature *F*, as shown in Equation (13). In this manner, the proposed hybrid framework provides more diversity in the estimation of class probability. The joint feature is fed into the fully connected layer for final classification.

(13) $$F=con[f_{s},f_{t}]$$

**Algorithm 1** Training process of the proposed framework.**Input:** PTB Dataset D={L,y}, Epoch *E*, Batch size *B* **Output:** The well-trained hybrid neural network Model
1:Split *D* into training set $D_{Tr}$, validation set $D_{Va}$ and testing set $D_{Te}$ in the proportion of 3:1:1;2:**while**(epoch≤E)**do**3:   **for** start in range $\left(0,length\left(D_{Tr}\right),B\right)$ **do**4:   end=start+B;5:   $batch=D_{Tr}\left[start:end\right]$;6:   **for** beat sample $L_{i}\in batch$ **do**7:     // Multi-lead Attention Module;8:     $\alpha_{1}=StepReLU\left(\tanh\left(W_{1}L_{i}+b_{1}\right)w_{1}\right)$;9:     $X_{i}=\alpha_{1}⊗L_{i}$;10:     // CNN with Attention Mechanism;11:     $C_{1}\leftarrow$ Conv2D($X_{i},kernels$); kernel size: (3,3), (5,1), and (7,1); each size has 20 kernels with one stride;12:     $C_{1}\leftarrow$ activation ($C_{1},ReLU$);13:     $C_{1}\leftarrow$ BatchNormalization($C_{1}$);14:     $C_{1}\leftarrow$ MaxPooling($C_{1},window$); the size of window is (2,2) with one stride;15:     $C_{1}\leftarrow$ Dropout($C_{1}$);16:     $C_{2}\leftarrow$ Conv2D($C_{1},kernels$); kernel size: (3,3), (5,1), and (7,1); each size has 20 kernels with one stride;17:     $C_{2}\leftarrow$ activation ($C_{2},ReLU$);18:     $C_{2}\leftarrow$ BatchNormalization($C_{2}$);19:     $C_{2}\leftarrow$ MaxPooling($C_{2},window$); the size of window is (2,2) with one stride;20:     $C_{2}\leftarrow$ Dropout($C_{2}$);21:     $C_{2}\leftarrow$ Reshape($C_{2}$);22:     Spatial features $f_{s}\leftarrow$ Attention($C_{2}$);23:     // BiGRU with Attention Mechanism;24:     $\vec{h_{t}}\leftarrow$ forward GRU($X_{i}$);25:     $\overset{\leftarrow }{h_{t}}\leftarrow$ backward GRU($X_{i}$);26:     $H_{t}\leftarrow$ concatenate($\vec{h_{t}},\overset{\leftarrow }{h_{t}}$);27:     $H_{t}\leftarrow$ BatchNormalization ($H_{t}$);28:     $H_{t}\leftarrow$ Dropout ($H_{t}$);29:     Temporal features $f_{t}\leftarrow$ Attention($H_{t}$);30:     // Merge and Classification;31:     Features F← concatenate($f_{s},f_{t}$);32:     F← BatchNormalization (*F*);33:     F← Dropout (*F*);34:     $y_{pre}\leftarrow$ FullyConnected(*F*);35:     **if** MI detection **then**36:       cross_entropy=binary_crossentropy;37:     **else if** MI location **then**38:       cross_entropy=categorical_crossentropy;39:     **end if**40:    **end for**41:    $loss=\frac{1}{B}\sum_{batch}{cross}_{-}entropy$ ($y_{true},y_{pre}$);42:    Training ← use AdamOptimizer to minimize loss;43:  **end for**44:  epoch+=1;45:**end while**46:**return** well-trained Model;


Attentive CNN module focuses more on the distinguishable neighbor information among different ECG leads, while BiGRU with attention mechanism is skilled at extracting essential temporal characteristics inside each lead. Obviously, the two modules complement each other to make the extracted features more comprehensive and efficient, thus achieving higher performance.

Compared with the hand-crafted features extracted by traditional classifiers, the end-to-end framework integrates the lead selection, feature extraction, feature reduction and MI classification as a whole system. Moreover, the creative and efficient feature processing structure can generate discriminative spatial and temporal features by co-training the two modules.

## 5. Results

### 5.1. Evaluation Metrics

The accuracy (Acc) of the classification is the proportion of correctly classified samples to the total number of samples. The classification accuracy measures the universal classification results, which is defined by true positive (TP), true negative (TN), false positive (FP), and false negative rates (FN) in Equation (14).

(14) $$Acc=\frac{TP+TN}{TP+FP+TN+FN}$$

Sensitivity (Sen) measures the proportion of real MI patients who are correctly classified, and defined as Equation (15). Instead, specificity (Spe), defined in Equation (16), measures the proportion of real healthy people who are correctly predicted. High sensitivity indicates low rate of missed diagnosis, i.e., few MI patients are classified as healthy individuals. High specificity indicates low rate of misdiagnosis, i.e., few healthy individuals are deemed as MI patients.

(15) $$Sen=\frac{TP}{TP+FN}$$

(16) $$Spe=\frac{TN}{FP+TN}$$

### 5.2. Experimental Methodology

**Figure 4:**
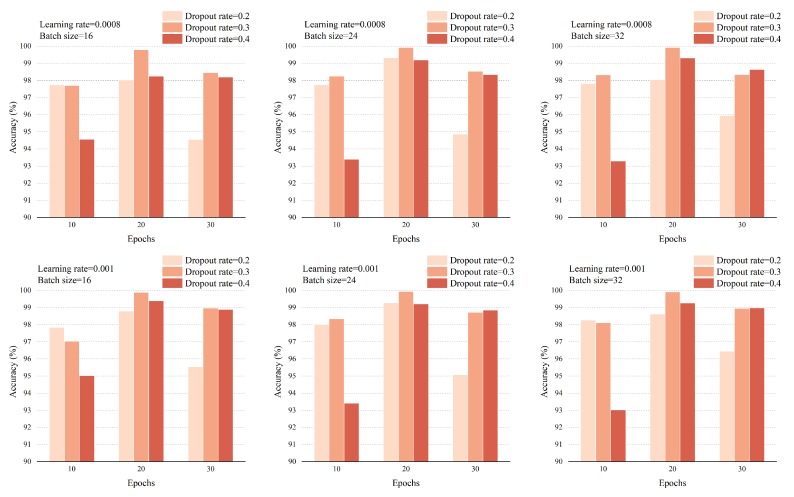
The adjustment and evaluation of the parameters.

Based on the PTB dataset, MI detection and MI location under both intra-patient and inter-patient schemes were implemented to verify the effectiveness of the proposed MLA-CNN-BiGRU framework. All the experiments were based on the evaluation of Acc, Sen, and Spe and experimental results were obtained by five-fold cross-validation. Under intra-patient scheme, the total beats were randomly divided into five approximately equal parts. For each iteration, three parts were used to train the model. One part was used as validation set to optimize the parameters of the framework. The remaining part was used as testing set to evaluate the final performance. As for the inter-patient scheme, patients were randomly separated in the proportion of 3:1:1 for training, validation, and testing, and the corresponding beats formed the training set, validation set, and testing set. Grid-search method was implemented to optimize parameters over a given parameter grid. By virtue of this technique, an exhaustive search over the value of a specified parameter was performed. Parameters including dropout rate, learning rate, batch size, and the number of epochs were selected by trial and error based on the validation set. The search range of dropout rate was set to be 0.2, 0.3, and 0.4. The options of learning rate were 0.0008 and 0.001. Batch size was set to be different in three cases, which equaled 16, 24, and 32. Additionally, the number of epochs was set to be 10, 20, and 30. The results of each search are shown in Figure 4. Moreover, to explore the effect of component structures in the proposed framework, ablation experiments were conducted based on MI detection. The proposed framework was also compared with one of the most popular dimensionality reduction method, i.e., PCA [48], combined with multi-layer perceptron (MLP) for classification (PCA-MLP). Then, MI location was conducted as application and extension of our framework. All the experiments were implemented with Windows 10 Operating System, NVIDIA GeForce GTX 1660 Ti GPU, Genuine Intel (R) Core (TM) i7-9700K CPU @ 3.60 GHz and 32 GB RAM. The program was carried out by TensorFlow-gpu 1.9.0 and Keras 2.2.4 with Python 3.6.5.

### 5.3. MI Detection

MI detection is a binary classification task to distinguish MI patients from HCs. The experiments were conducted on 80 12-lead ECG records from HCs and 368 records from MI patients with a total of 760,128 beats. Moreover, ablation experiments based on component structures were conducted with the same parameters as the MLA-CNN-BiGRU framework. In detail, the ablation structures were MLA-BiGRU module without feature attention mechanism (MLA-BiGRU${}_{w/o}$), MLA-CNN module without feature attention mechanism (MLA-CNN${}_{w/o}$), MLA-BiGRU module with feature attention mechanism (MLA-BiGRU), MLA-CNN module with feature attention mechanism (MLA-CNN), and CNN-BiGRU without MLA mechanism but with feature attention mechanism (CNN-BiGRU). Additionally, PCA-MLP was tested as a comparative framework that integrated the most popular dimensionality reduction method with a basic neural network.

#### 5.3.1. Intra-Patient Scheme

**Figure 5:**
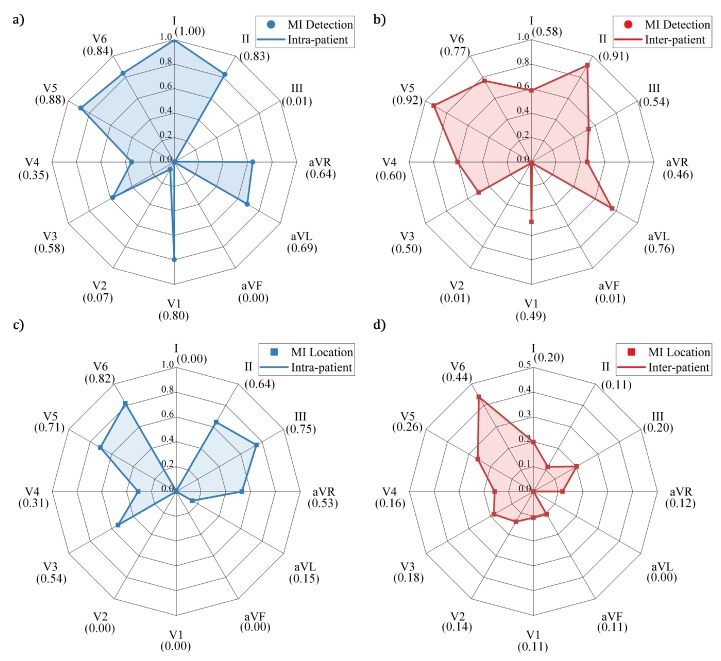
Lead weights obtained by five-fold cross-validation: (**a**) lead weights in MI detection under intra-scheme; (**b**) lead weights in MI detection under inter-scheme; (**c**) lead weights in MI location under intra-scheme; and (**d**) lead weights in MI location under inter-scheme.

In MI detection under intra-patient scheme, the results of ablation experiments are demonstrated in Table 2, and those of the comparative experiment are shown in Table 3. The average values of the lead weights obtained by five-fold cross-validation are presented in Figure 5a. Experimental results indicate that, among all the component structures, the proposed MLA-CNN-BiGRU achieved the highest average Acc of 99.93%, Sen of 99.99%, and Spe of 99.63%. Simultaneously, the proposed framework also obtained the lowest standard deviation (std) of the three metrics, i.e., 0.05%, 0.004%, and 0.31%, respectively. The results of MLA-BiGRU${}_{w/o}$ were comparable to MLA-CNN${}_{w/o}$ but worse than MLA-BiGRU. MLA-CNN achieved better performance than CNN-BiGRU and MLA-BiGRU, but was still inferior to MLA-CNN-BiGRU. When comparing with PCA-MLP, the proposed framework maintained the highest overall performance as well. According to Figure 5a, the highly recommended leads are I, II, V5, and V6, all of which have weights in excess of 0.8. Lead aVF is entirely excluded because its weight is zero.

#### 5.3.2. Inter-Patient Scheme

As for MI detection under inter-patient scheme, the results of ablation experiments are summarized in Table 4, and the results of the comparison experiment are given in Table 3. The average lead weights are illustrated in Figure 5b. According to the experimental results, the proposed framework achieved highest average Acc of 96.50%, Sen of 97.10%, and Spe of 93.34% among all the methods. The proposed framework also obtained the lowest std in Acc and Spe, i.e., 2.25% and 4.84%, respectively. Consistent with the intra-patient scheme, MLA-CNN achieved superior performance to MLA-BiGRU${}_{w/o}$, MLA-CNN${}_{w/o}$, MLA-BiGRU, and CNN-BiGRU, but was still worse than the complete hybrid framework MLA-CNN-BiGRU. Compared with PCA-MLP in Table 3, the Acc of the proposed framework was improved by 24.88%, and its std was low. As indicated in Figure 5b, the leads with large weights are II, aVL, V5, and V6, all with weights above 0.7. Leads aVF and V2 are virtually redundant and ineffective.

### 5.4. MI Location

MI location is a multi-class classification task. In this study, the proposed MLA-CNN-BiGRU framework was applied for MI location based on six classes of 12-lead ECG records, namely HC and five types of MI. In detail, the six categories of data were comprised of 80 records from HCs, 47 records from AMI, 43 records from ALMI, 79 records from ASMI, 89 records from IMI, and 56 records from ILMI, with a total of 678,612 beats.

#### 5.4.1. Intra-Patient Scheme

MI location under intra-patient scheme was performed. The results of five-fold cross-validation are presented in Table 5, including the metrics calculated for each category. The average values of the lead weights obtained by cross validation are shown in Figure 5c. As presented in Table 5, MLA-CNN-BiGRU achieved the average Acc of 99.11%, Sen of 99.02%, and Spe of 99.10%. According to Figure 5c, the recommended leads for MI location are II, III, V5, and V6, all with weights over 0.6. Leads I, aVF, V1, and V2 are precluded for their few contributions to the subsequent processing.

#### 5.4.2. Inter-Patient Scheme

For inter-patient scheme, Table 5 demonstrates the results of five-fold cross-validation. The average lead weights are illustrated in Figure 5d. As can be observed in Table 5, the experimental results in this case are much lower than those in the other three cases. In addition, lead weights were relatively small, with V6 having a maximum lead weight of 0.44. Only lead aVL was eliminated during the training process of the model. Due to the uneven distribution of beats numbers, the category with the highest performance in different folds varied considerably.

## 6. Discussion

This paper presents a novel and reliable MLA-CNN-BiGRU framework for MI detection and location under both intra-patient scheme and inter-patient scheme. Meanwhile, elaborate ablation experiments based on MLA mechanism, CNN module, BiGRU module, and feature integration attention mechanism were carried out. The ablation experiments aimed to explore the role of the component structure in improving the performance of MI diagnosis. Moreover, the proposed framework was compared with another widely adopted feature extraction method. Standard metrics, i.e., Acc, Sen, and Spe, were employed to verify the effectiveness of the proposed framework. Among all the experiments presented in Section 5, MLA-CNN-BiGRU performed best by comparing different components and another feature extraction method in MI diagnosis under both intra-patient and inter-patient schemes.

As shown in Figure 4, the accuracy is almost identical when the batch size equals 24 and 32, and slightly lower when the batch size equals 16. The performance with a learning rate of 0.001 was slightly better than that with a learning rate of 0.0008. It was most suitable to set the number of epochs to 20. Insufficient number of epochs led to the under-fitting of the neural network. On the contrary, excessive training rounds gave rise to the problem of over-fitting. The dropout rate also exerted influence on the accuracy and therefore it could not be set too high or too low. A dropout rate of 0.3 was more appropriate.

In this study, the rank (from high to low) of the lead contribution of MI detection is: I, V5, V6, II, V1, aVL, aVR, V3, V4, V2, III, and aVF under intra-patient scheme; and V5, II, V6, aVL, V4, I, III, V3, V1, aVR, V2, and aVF under inter-patient scheme. The rank (from high to low) of the lead contribution of MI location is: V6, III, V5, II, V3, aVR, V4, aVL, I, aVF, V1, and V2 under intra-patient scheme; and V6, V5, I, III, V3, V4, V2, aVR, II, aVF, V1, and aVL under inter-patient scheme. In theory, each lead reflects a different perspective of the heart activity. More precisely, leads V3 and V4 correspond to the anterior aspect of the heart. Leads V1 and V2 reflect both septal and posterior aspects of the heart. Inferior part is related to leads II, III, and aVF. Lateral part is associated with leads I, aVL, V5, and V6. Lead aVR is related to the endocardial part [49]. From the experimental results, leads I, II, III, V3, V5, and V6 were of more importance, which may be caused by data distribution. Since most of the MIs in the PTB dataset were related to anterior, inferior, and lateral parts, the weights were primarily assigned to the leads that could assist in the diagnosis of these three main parts. In the literature, lead V5 achieved the highest sensitivity in detecting myocardial ischemia [50] and presented the best performance among all the 12 leads of ECG signals [51]. In addition, lead II is a commonly used lead for basic cardiac monitoring [19]. As shown in Table 6, leads I, III, and V3 were also selected and achieved good results. The previous research is consistent with our experimental results that leads V5 and II made a greater contribution. In fact, the lead contribution is not only related to the model architecture, but also to the sample distribution. It should be mentioned that this study did not focus on which leads were closely related to MI diagnosis from pathology. Specifically, this study contributes to optimizing the number of leads by selecting the most essential ones, which can assist the proposed framework to obtain the most effective diagnosis.

Neural networks are good at processing high-dimensional nonlinear data by virtue of automatic feature extraction. Compared with PCA presented in Table 3, the neural network frameworks have superior performance because the feature extraction and classification processes of neural networks are end-to-end systems. CNN and GRU are capable of extracting various features directly from original data through convolutional abstraction and gate-based memory cells. CNNs are popular models for image data processing, while GRUs are familiar with processing temporal sequence data. Compared with BiGRU module, CNN module has better performance, as shown in both Table 2 and Table 4. It indicates that spatial features contained more useful information for the diagnosis of MI. Additionally, the component structure with attention layer was better than that without attention mechanism. It indicates that there remained redundant information after the feature extraction, which required the attention layer for effective integration. After eliminating the MLA layer, as shown in Table 2 and Table 4, the performance of CNN-BiGRU is lower than that of the complete framework, which can verify the effectiveness of the proposed MLA mechanism. Furthermore, the hybrid framework had superior performance and stability to the component structures. Despite involving additional training process, the combination of spatial and temporal features with attention mechanism exhibited more robust performance in comparison with other methods. The combined features were deemed to be discriminative in the diagnosis of MI. It was essential to consider relationships between different leads and the temporal characteristics of ECG signals.

MI diagnosis is composed of detection and location in this study. MI detection is a binary classification problem, while MI location is a six-class multi-classification task. The results indicate that MI detection obtained better performance than MI location and intra-patient scheme achieved better performance than inter-patient scheme. Since the inter-patient scheme could prevent training and testing the model using the beats from the same patients, it exerted more difficulties on the model to overcome the individual difference. Furthermore, the inter-patient scheme caused the unbalanced distribution of data and greatly affected the performance of the model. Notably, the performance of MI location under inter-patient scheme remains to be improved.

The proposed framework was compared with previous studies on the same PTB dataset, as shown in Table 6. Among all the methods, the proposed framework achieved highest accuracy in MI detection under both the intra-patient scheme and inter-patient scheme. Compared with the method of Han and Shi [24], the accuracy, sensitivity, and specificity of our framework were improved by 7.20%, 16.39%, and 7.63%, respectively in MI location under inter-patient scheme. Moreover, this study has several merits, such as the utilization of 12-lead ECG signals, the effective end-to-end system, the selection of leads based on model-driven approach, elaborate feature extraction from both spatial and temporal perspectives of the signals, and exhaustive experiments among MI detection and location under two schemes. Furthermore, our study designed ablation experiments to examine the effectiveness of component structures, which more comprehensively verified the reliability of the proposed framework.

The proposed framework achieved the optimal results; however, there are three limitations that need to be improved in our future work. Firstly, although it is worthwhile to make sacrifices on training time and memory storage to achieve higher diagnostic accuracy, the proposed hybrid framework has complicated structure and extensive parameters. It exerts challenge on embedding the network into mobile portable devices. The architecture of the network therefore remains to be explored and further optimized. For instance, it is very effective to optimize the BiGRU module that has a slow operating speed. Additionally, the parameters of attention mechanism should be reduced appropriately. In essence, these changes are the trade-offs between the complexity and accuracy of the framework, which deserve elaboration in the future study. Secondly, to achieve expert diagnosis, the process of lead selection should be explained more precisely by comparative experiments and pathological analysis. Thirdly, the framework should be evaluated on more datasets with diversity to confirm the robustness in practical applications.

## 7. Conclusions

In this paper, a novel MLA-CNN-BiGRU framework for automatic MI detection and location is presented based on 12-lead ECG signals. To efficiently and effectively employ all 12 leads, the MLA mechanism is developed to weight the contribution of each lead by the designed activation function, and useful leads can be selected for the subsequent process. In the process of feature extraction, CNN is introduced to extract spatial features from inter-correlated ECG signals among the different leads. Meanwhile, BiGRU is applied to extract temporal features inside each lead. Both neural networks have an attention layer in the end for feature integration. Then, the spatial and temporal features extracted from two modules are combined as global spatial-temporal features for the final classification process. Comparative and ablation experiments were conducted under inter-patient and intra-patient schemes to confirm the effectiveness of the proposed framework in MI detection and location. The experimental results indicate that the proposed framework demonstrated satisfactory performance on the PTB dataset, but the location under inter-patient scheme needs further improvement. With the proposed model-based approach, this study serves as a preliminary exploration on the importance evaluation of each lead in the diagnosis of MI. Moreover, in the field of 12-lead ECG signal processing, this study provides a new insight into the application of attention mechanism and parallel feature extraction structure based on deep learning.

## Figures and Tables

**Table 1:** Summary of Physikalisch-Technische Bundesanstalt (PTB) dataset in this study.

Class	No. of Records	No. of 12-Lead Beats
AMI	47	81,168
ALMI	43	80,988
ASMI	79	140,256
IMI	89	151,716
ILMI	56	97,296
Other MIs	54	81,516
HCs	80	127,188
Total	448	760,128

Anterior myocardial infarction (AMI); Antero-lateral myocardial infarction (ALMI); Antero-septal myocardial infarction (ASMI); Inferior myocardial infarction (IMI); Inferolateral myocardial infarction (ILMI); Myocardial infarction (MI); Healthy control (HC).

**Table 2:** Ablation experiments of MI detection by five-fold cross-validation under intra-patient scheme.

MLA-BiGRU${}_{w/o}$	Acc (%)	Sen (%)	Spe (%)	MLA-CNN${}_{w/o}$	Acc (%)	Sen (%)	Spe (%)
Fold 1	86.90	96.06	41.32	Fold 1	91.81	97.28	64.62
Fold 2	91.58	96.19	68.56	Fold 2	92.16	98.80	59.05
Fold 3	93.46	97.97	70.75	Fold 3	93.41	96.31	78.77
Fold 4	87.84	96.62	45.30	Fold 4	92.94	99.29	62.18
Fold 5	96.24	97.37	90.71	Fold 5	87.51	99.34	29.37
Mean	91.20	96.84	63.33	Mean	91.57	98.20	58.80
Std	3.89	0.81	20.26	Std	2.35	1.35	18.10
**MLA-BiGRU**	**Acc (%)**	**Sen (%)**	**Spe (%)**	**MLA-CNN**	**Acc (%)**	**Sen (%)**	**Spe (%)**
Fold 1	96.43	98.29	87.17	Fold 1	93.91	100.00	63.63
Fold 2	83.31	100.00	0.00	Fold 2	91.69	99.75	51.44
Fold 3	95.50	99.13	77.19	Fold 3	99.61	99.80	98.66
Fold 4	99.62	99.61	99.68	Fold 4	99.73	99.99	98.48
Fold 5	91.94	91.91	92.11	Fold 5	99.84	99.88	99.67
Mean	93.36	97.79	71.23	Mean	96.96	99.88	82.38
Std	6.25	3.35	40.65	Std	3.88	0.11	23.09
**CNN-BiGRU**	**Acc (%)**	**Sen (%)**	**Spe (%)**	**MLA-CNN-BiGRU**	**Acc (%)**	**Sen (%)**	**Spe (%)**
Fold 1	97.64	99.31	89.34	Fold 1	99.93	99.99	99.62
Fold 2	98.27	99.46	92.34	Fold 2	99.85	100.00	99.10
Fold 3	98.31	99.70	91.32	Fold 3	99.95	99.99	99.76
Fold 4	91.06	99.26	51.34	Fold 4	99.96	99.99	99.82
Fold 5	93.54	98.92	67.09	Fold 5	99.97	99.99	99.86
Mean	95.76	99.33	78.29	Mean	**99.93**	**99.99**	**99.63**
Std	3.29	0.29	18.31	Std	0.05	0.004	0.31

Best performance is highlighted in bold.

**Table 3:** Comparative experiments of MI detection by five-fold cross-validation.

Framework	Intra-Patient Scheme				Inter-Patient Scheme			
	Folds	Acc (%)	Sen (%)	Spe (%)	Folds	Acc (%)	Sen (%)	Spe (%)
PCA-MLP	Fold 1	72.45	85.70	6.56	Fold 1	79.38	91.43	25.16
	Fold 2	76.61	89.85	10.54	Fold 2	54.11	76.32	12.13
	Fold 3	74.69	86.90	13.07	Fold 3	68.72	81.25	0.76
	Fold 4	89.72	97.16	53.69	Fold 4	78.70	84.79	0.00
	Fold 5	91.48	96.96	64.52	Fold 5	77.20	91.47	0.00
	Mean	80.99	91.31	29.68	Mean	71.62	85.05	7.61
	Std	8.92	5.46	27.24	Std	10.68	6.57	11.08
MLA-CNN-BiGRU	Mean	**99.93**	**99.99**	**99.63**	Mean	**96.50**	**97.10**	**93.34**
	Std	0.05	0.004	0.31	Std	2.25	2.60	4.84

Best performance is highlighted in bold.

**Table 4:** Ablation experiments of MI detection by five-fold cross-validation under inter-patient scheme.

MLA-BiGRU${}_{w/o}$	Acc (%)	Sen (%)	Spe (%)	MLA-CNN${}_{w/o}$	Acc (%)	Sen (%)	Spe (%)
Fold 1	80.98	92.15	30.71	Fold 1	87.04	94.23	54.73
Fold 2	87.11	83.95	93.10	Fold 2	85.99	86.68	84.70
Fold 3	85.56	92.62	47.25	Fold 3	85.74	99.88	9.02
Fold 4	92.52	95.86	49.41	Fold 4	91.31	92.09	81.24
Fold 5	84.40	100.00	0.00	Fold 5	90.70	97.21	55.47
Mean	86.11	92.92	44.09	Mean	88.16	94.02	57.03
Std	4.23	5.91	33.78	Std	2.65	5.05	30.27
**MLA-BiGRU**	**Acc (%)**	**Sen (%)**	**Spe (%)**	**MLA-CNN**	**Acc (%)**	**Sen (%)**	**Spe (%)**
Fold 1	84.83	94.54	41.11	Fold 1	90.47	99.97	47.72
Fold 2	89.59	84.24	99.69	Fold 2	93.83	94.34	92.85
Fold 3	84.44	100.00	0.00	Fold 3	95.59	100.00	71.65
Fold 4	93.20	99.97	5.70	Fold 4	93.07	99.99	3.68
Fold 5	86.19	99.99	11.52	Fold 5	99.90	100.00	99.36
Mean	87.65	95.75	31.60	Mean	94.57	98.86	63.05
Std	3.71	6.85	41.23	Std	3.50	2.53	38.86
**CNN-BiGRU**	**Acc (%)**	**Sen (%)**	**Spe (%)**	**MLA-CNN-BiGRU**	**Acc (%)**	**Sen (%)**	**Spe (%)**
Fold 1	93.69	95.71	84.58	Fold 1	92.93	93.70	89.48
Fold 2	97.29	98.59	94.84	Fold 2	95.59	95.20	96.33
Fold 3	88.97	99.97	29.25	Fold 3	97.93	98.92	92.55
Fold 4	96.18	96.61	90.62	Fold 4	97.87	97.70	100.00
Fold 5	86.07	99.97	10.89	Fold 5	98.17	99.98	88.36
Mean	92.44	98.17	62.04	Mean	**96.50**	**97.10**	**93.34**
Std	4.79	1.95	39.03	Std	2.25	2.60	4.84

Best performance is highlighted in bold.

**Table 5:** Results on MI location by five-fold cross-validation.

Folds	Category	Intra-patient Scheme			Inter-patient Scheme		
		Acc (%)	Sen (%)	Spe (%)	Acc (%)	Sen (%)	Spe (%)
Fold 1	AMI	98.13	99.70	97.93	62.06	78.51	59.31
	ALMI	98.13	96.97	98.30	62.06	22.78	66.05
	ASMI	98.13	93.64	99.29	62.06	58.90	63.02
	IMI	98.13	99.80	97.65	62.06	41.64	66.28
	ILMI	98.13	99.38	97.93	62.06	58.18	62.93
	HC	98.13	99.86	97.74	62.06	97.24	54.51
	Mean	**98.13**	**98.22**	**98.14**	**62.06**	**59.54**	**62.02**
Fold 2	AMI	98.07	93.74	98.64	58.61	39.87	61.20
	ALMI	98.07	95.81	98.38	58.61	54.53	58.86
	ASMI	98.07	97.05	98.34	58.61	35.45	65.90
	IMI	98.07	99.76	97.58	58.61	82.28	52.59
	ILMI	98.07	99.82	97.78	58.61	67.09	56.43
	HC	98.07	99.95	97.64	58.61	67.19	56.79
	Mean	**98.07**	**97.69**	**98.06**	**58.61**	**57.74**	**58.63**
Fold 3	AMI	99.73	99.78	99.72	46.19	89.88	39.87
	ALMI	99.73	98.59	99.88	46.19	99.68	44.66
	ASMI	99.73	99.96	99.67	46.19	12.77	65.39
	IMI	99.73	99.88	99.68	46.19	72.31	42.72
	ILMI	99.73	99.75	99.72	46.19	34.19	48.60
	HC	99.73	99.95	99.67	46.19	67.29	41.04
	Mean	**99.73**	**99.65**	**99.72**	**46.19**	**62.69**	**47.05**
Fold 4	AMI	99.85	99.78	99.86	72.68	72.64	72.69
	ALMI	99.85	99.79	99.86	72.68	65.10	74.03
	ASMI	99.85	99.96	99.82	72.68	46.56	75.98
	IMI	99.85	99.80	99.86	72.68	81.03	69.84
	ILMI	99.85	99.75	99.87	72.68	95.48	70.34
	HC	99.85	99.95	99.82	72.68	71.59	72.85
	Mean	**99.85**	**99.84**	**99.85**	**72.68**	**72.07**	**72.62**
Fold 5	AMI	99.75	99.78	99.75	75.18	57.96	78.53
	ALMI	99.75	99.54	99.78	75.18	100.00	74.13
	ASMI	99.75	100.00	99.69	75.18	69.05	75.91
	IMI	99.75	99.96	99.69	75.18	93.48	66.01
	ILMI	99.75	99.15	99.86	75.18	2.28	81.78
	HC	99.75	99.81	99.74	75.18	83.99	71.86
	Mean	**99.75**	**99.71**	**99.75**	**75.18**	**67.79**	**74.70**
**five-fold Mean**	\	**99.11**	**99.02**	**99.10**	**62.94**	**63.97**	**63.00**

Average values are highlighted in bold.

**Table 6:** Comparison of frameworks for MI detection and location by ECG signals on the PTB dataset.

Year	Lead*	Records or Beats	Dataset	Framework	Detection	Location	Performance	
							Intra-Patient	Inter-Patient
2016 [51]	Lead 11 for detection (V5) Lead 9 for location (V3)	Beats	485,753 MI 125,652 HC	DWT + KNN	✓	✓	Detection: Acc = 98.80% Sen = 99.45% Spe = 96.27% Location: Acc = 98.74% Sen = 99.55% Spe = 99.16%	No
2017 [12]	Lead 2 (II)	Beats	40,182 MI 10,546 HC	FAWT and SEnt + LS-SVM	✓	×	Acc = 99.31% Sen = 99.62% Spe = 98.12%	No
2017 [19]	Lead 2 (II)	Beats	40,182 MI 10,546 HC	CNN	✓	×	Acc = 95.22% Sen = 95.49% Spe = 94.19%	No
2017 [20]	Lead 5, 8, 9 and 11 (aVL, V2, V3 and V5)	Beats	167 MI records 80 HC records	ML-CNN	✓	×	Acc = 96.00% Sen = 95.40% Spe = 97.37%	No
2018 [3]	Lead 2,3 and 8 (II, III, and V2)	Beats	15,000 MI 5000 HC	Handcrafted features + LR	✓	×	Acc = 95.60% Sen = 96.50% Spe = 92.70%	No
2018 [21]	Lead 1 (I)	Records	368 MI 80 HC 74 Other 278 Noisy	CNN-LSTM stacking decoding	✓	×	No	Sen = 92.4% Spe = 97.7%
2019 [22]	12 Leads	Records	369 MI 79 HC	BiLSTM Heartbeat-attention	✓	×	No	Acc = 94.77% Sen = 95.58% Spe = 90.48%
2019 [25]	12 Leads	Beats	28,213 MI 5373 HC	MODWPT + PCA + SVM (Intra) MODWPT + PCA + Bagging (Inter)	✓	×	Acc = 99.75% Sen = 99.37% Spe = 99.37%	Acc = 92.69% Sen = 80.96% Spe = 80.96%
2019 [23]	12 Leads	Beats	53,712 MI 10,638 HC	CNN + BiLSTM	✓	×	Acc = 99.90% Sen = 99.97% Spe = 99.54%	Acc = 93.08% Sen = 94.42% Spe = 86.29%
2019 [24]	12 Leads	Beats	28,213MI 5373 HC	ML-ResNet	✓	✓	Detection: Acc = 99.92% Sen = 99.98% Spe = 99.77% Location: Acc = 99.72% Sen = 99.63% Spe = 99.72%	Detection: Acc = 95.49% Sen = 94.85% Spe = 97.37% Location: Acc = 55.74% Sen = 47.58% Spe = 55.37%
**Proposed**	**12 Leads**	**Beats**	**632,940 MI** **127,188 HC**	**MLA-CNN-BiGRU**	**✓**	**✓**	**Detection:** **Acc = 99.93%** **Sen = 99.99%** **Spe = 99.63%** **Location:** **Acc = 99.11%** **Sen = 99.02%** **Spe = 99.10%**	**Detection:** **Acc = 96.50%** **Sen = 97.10%** **Spe = 93.34%** **Location:** **Acc = 62.94%** **Sen = 63.97%** **Spe = 63.00%**

Lead*: The leads that get the best results. Discrete wavelet transform (DWT); K-nearest neighbours (KNN); Flexible analytic wavelet transform and Sample entropy (FAWT and SEnt); Least-squares support vector machine (LS-SVM); Logistic regression (LR); Maximal overlap discrete wavelet packet transform (MODWPT); Principal component analysis (PCA); Multi-lead residual neural network (ML-ResNet); Multilead-CNN (ML-CNN); Bidirectional Long Short Term Memory (BiLSTM).

## References

[1] Benjamin E.J., Muntner P., Bittencourt M.S. (2019). Heart disease and stroke statistics—2019 update: A report from the American Heart Association. Circulation.

[2] Thygesen K., Alpert J.S., Jaffe A.S., Chaitman B.R., Bax J.J., Morrow D.A., White H.D., The Executive Group on behalf of the Joint European Society of Cardiology (ESC), American College of Cardiology (ACC), American Heart Association (AHA) (2018). Fourth universal definition of myocardial infarction (2018). J. Am. Coll. Cardiol..

[3] Sadhukhan D., Pal S., Mitra M. (2018). Automated identification of myocardial infarction using harmonic phase distribution pattern of ECG data. IEEE Trans. Instrum. Meas..

[4] Liu B., Liu J., Wang G., Huang K., Li F., Zheng Y., Luo Y., Zhou F. (2015). A novel electrocardiogram parameterization algorithm and its application in myocardial infarction detection. Comput. Biol. Med..

[5] Mixon T.A., Suhr E., Caldwell G., Greenberg R.D., Colato F., Blackwell J., Jo C.H., Dehmer G.J. (2012). Retrospective description and analysis of consecutive catheterization laboratory ST-segment elevation myocardial infarction activations with proposal, rationale, and use of a new classification scheme. Circ. Cardiovasc. Qual. Outcomes.

[6] Faust O., Acharya U.R., Tamura T. (2012). Formal design methods for reliable computer-aided diagnosis: A review. IEEE Rev. Biomed. Eng..

[7] Lu H., Ong K., Chia P. An automated ECG classification system based on a neuro-fuzzy system. Proceedings of the Computers in Cardiology 2000.

[8] Ansari S., Farzaneh N., Duda M., Horan K., Andersson H.B., Goldberger Z.D., Nallamothu B.K., Najarian K. (2017). A review of automated methods for detection of myocardial ischemia and infarction using electrocardiogram and electronic health records. IEEE Rev. Biomed. Eng..

[9] Barmpoutis P., Dimitropoulos K., Apostolidis A., Grammalidis N. (2019). Multi-lead ECG signal analysis for myocardial infarction detection and localization through the mapping of Grassmannian and Euclidean features into a common Hilbert space. Biomed. Signal Process. Control.

[10] Banerjee S., Mitra M. (2013). Application of cross wavelet transform for ECG pattern analysis and classification. IEEE Trans. Instrum. Meas..

[11] Acharya U.R., Fujita H., Adam M., Lih O.S., Sudarshan V.K., Hong T.J., Koh J.E., Hagiwara Y., Chua C.K., Poo C.K. (2017). Automated characterization and classification of coronary artery disease and myocardial infarction by decomposition of ECG signals: A comparative study. Inf. Sci..

[12] Kumar M., Pachori R., Acharya U. (2017). Automated diagnosis of myocardial infarction ECG signals using sample entropy in flexible analytic wavelet transform framework. Entropy.

[13] Sharma L., Tripathy R., Dandapat S. (2015). Multiscale energy and eigenspace approach to detection and localization of myocardial infarction. IEEE Trans. Biomed. Eng..

[14] Faust O., Hagiwara Y., Hong T.J., Lih O.S., Acharya U.R. (2018). Deep learning for healthcare applications based on physiological signals: A review. Comput. Methods Programs Biomed..

[15] Lu B., Fu L., Nie B., Peng Z., Liu H. (2019). A Novel Framework with High Diagnostic Sensitivity for Lung Cancer Detection by Electronic Nose. Sensors.

[16] Yuan Y., Jia K. (2019). FusionAtt: Deep Fusional Attention Networks for Multi-Channel Biomedical Signals. Sensors.

[17] LeCun Y., Bengio Y., Hinton G. (2015). Deep learning. Nature.

[18] Zhang W., Yang D., Wang H., Huang X., Gidlund M. (2019). CarNet: A Dual Correlation Method for Health Perception of Rotating Machinery. IEEE Sens. J..

[19] Acharya U.R., Fujita H., Oh S.L., Hagiwara Y., Tan J.H., Adam M. (2017). Application of deep convolutional neural network for automated detection of myocardial infarction using ECG signals. Inf. Sci..

[20] Liu W., Zhang M., Zhang Y., Liao Y., Huang Q., Chang S., Wang H., He J. (2017). Real-time multilead convolutional neural network for myocardial infarction detection. IEEE J. Biomed. Health Informat..

[21] Lui H.W., Chow K.L. (2018). Multiclass classification of myocardial infarction with convolutional and recurrent neural networks for portable ECG devices. Informat. Med. Unlocked.

[22] Zhang Y., Li J. (2019). Application of Heartbeat-Attention Mechanism for Detection of Myocardial Infarction Using 12-Lead ECG Records. Appl. Sci..

[23] Liu W., Wang F., Huang Q., Chang S., Wang H., He J. (2019). MFB-CBRNN: A hybrid network for MI detection using 12-lead ECGs. IEEE J. Biomed. Health Inform..

[24] Han C., Shi L. (2020). ML–ResNet: A novel network to detect and locate myocardial infarction using 12 leads ECG. Comput. Methods Programs Biomed..

[25] Han C., Shi L. (2019). Automated interpretable detection of myocardial infarction fusing energy entropy and morphological features. Comput. Methods Programs Biomed..

[26] Itti L., Koch C. (2001). Computational modelling of visual attention. Nat. Rev. Neurosci..

[27] Vaswani A., Shazeer N., Parmar N., Uszkoreit J., Jones L., Gomez A.N., Kaiser Ł., Polosukhin I. Attention is all you need. Proceedings of the Advances in Neural Information Processing Systems 30.

[28] Lin Z., Feng M., Santos C.N., Yu M., Xiang B., Zhou B., Bengio Y. A structured self-attentive sentence embedding. Proceedings of the 5th International Conference on Learning Representations.

[29] Huang T., Deng Z.H., Shen G., Chen X. (2020). A Window-Based Self-Attention approach for sentence encoding. Neurocomputing.

[30] LeCun Y., Bottou L., Bengio Y., Haffner P. (1998). Gradient-based learning applied to document recognition. Proc. IEEE.

[31] Yamashita R., Nishio M., Do R.K.G., Togashi K. (2018). Convolutional neural networks: an overview and application in radiology. Insights Imaging.

[32] Gu J., Wang Z., Kuen J., Ma L., Shahroudy A., Shuai B., Liu T., Wang X., Wang G., Cai J. (2018). Recent advances in convolutional neural networks. Pattern Recognit..

[33] Ioffe S., Szegedy C. Batch normalization: Accelerating deep network training by reducing internal covariate shift. Proceedings of the 32th International Conference on Machine Learning.

[34] Hinton G.E., Srivastava N., Krizhevsky A., Sutskever I., Salakhutdinov R.R. (2012). Improving neural networks by preventing co-adaptation of feature detectors. arXiv.

[35] Liu N., Wang L., Chang Q., Xing Y., Zhou X. (2018). A Simple and Effective Method for Detecting Myocardial Infarction Based on Deep Convolutional Neural Network. J. Med. Imaging Health Informat..

[36] Baloglu U.B., Talo M., Yildirim O., San Tan R., Acharya U.R. (2019). Classification of myocardial infarction with multi-lead ECG signals and deep CNN. Pattern Recognit. Lett..

[37] Liu W., Huang Q., Chang S., Wang H., He J. (2018). Multiple-feature-branch convolutional neural network for myocardial infarction diagnosis using electrocardiogram. Biomed. Signal Process. Control.

[38] Hochreiter S., Schmidhuber J. (1997). Long short-term memory. Neural Comput..

[39] Cho K., Van Merriënboer B., Bahdanau D., Bengio Y. (2014). On the properties of neural machine translation: Encoder-decoder approaches. arXiv.

[40] Bahdanau D., Cho K., Bengio Y. (2014). Neural machine translation by jointly learning to align and translate. arXiv.

[41] Chen J., Jiang D., Zhang Y. (2019). A Hierarchical Bidirectional GRU Model With Attention for EEG-Based Emotion Classification. IEEE Access.

[42] Lynn H.M., Pan S.B., Kim P. (2019). A Deep Bidirectional GRU Network Model for Biometric Electrocardiogram Classification Based on Recurrent Neural Networks. IEEE Access.

[43] PhysioBank P. (2000). PhysioNet: Components of a new research resource for complex physiologic signals. Circulation.

[44] Martis R.J., Acharya U.R., Min L.C. (2013). ECG beat classification using PCA, LDA, ICA and discrete wavelet transform. Biomed. Signal Process. Control.

[45] Pan J., Tompkins W.J. (1985). A real-time QRS detection algorithm. IEEE Trans. Biomed. Eng..

[46] Jing L., Wang T., Zhao M., Wang P. (2017). An adaptive multi-sensor data fusion method based on deep convolutional neural networks for fault diagnosis of planetary gearbox. Sensors.

[47] Boureau Y.L., Ponce J., LeCun Y. A theoretical analysis of feature pooling in visual recognition. Proceedings of the 27th International Conference on Machine Learning (ICML-10).

[48] Zhang G., Tang L., Zhou L., Liu Z., Liu Y., Jiang Z. (2019). Principal Component Analysis Method with Space and Time Windows for Damage Detection. Sensors.

[49] Chang P.C., Lin J.J., Hsieh J.C., Weng J. (2012). Myocardial infarction classification with multi-lead ECG using hidden Markov models and Gaussian mixture models. Appl. Soft Comput..

[50] Crawford M.H., Bernstein S.J., Deedwania P.C., DiMarco J.P., Ferrick K.J., Garson A., Green L.A., Greene H.L., Silka M.J., Stone P.H. (1999). ACC/AHA Guidelines for Ambulatory Electrocardiography: Executive Summary and Recommendations. Circulation.

[51] Acharya U.R., Fujita H., Sudarshan V.K., Oh S.L., Adam M., Koh J.E., Tan J.H., Ghista D.N., Martis R.J., Chua C.K. (2016). Automated detection and localization of myocardial infarction using electrocardiogram: A comparative study of different leads. Knowl.-Based Syst..

